# Toxicological assessment of nanocrystalline metal alloys with potential applications in the aeronautical field

**DOI:** 10.1038/s41598-022-05406-5

**Published:** 2022-01-27

**Authors:** Carlos Rumbo, Alvise Bianchin, Antonio Mario Locci, Rocío Barros, Sonia Martel Martín, Juan Antonio Tamayo-Ramos

**Affiliations:** 1 International Research Center in Critical Raw Materials-ICCRAM, Universidad de Burgos, Plaza Misael Bañuelos s/n, 09001 Burgos, Spain; 2 MBN Nanomaterialia S.P.A, 31050 Vascon Di Carbonera, TV Italy; 3 Dipartimento Di Ingegneria Meccanica, Chimica, e dei Materiali, Università Degli Studi Di Cagliari, via Marengo 2, 09123 Cagliari, Italy

**Keywords:** Environmental impact, Cell biology, Microbiology

## Abstract

The development of new candidate alloys with outstanding characteristics for their use in the aeronautical field is one of the main priorities for the sector. In this context, nanocrystaline (nc) alloys are considered relevant materials due to their special features, such as their exceptional physical and mechanical properties. However, another important point that needs to be considered with newly developed alloys is the potential toxicological impact that these materials may have in humans and other living organisms. The aim of this work was to perform a preliminary toxicological evaluation of three nc metal alloys (WCu, WAl and TiAl) in powder form produced by mechanical alloying, applying different in vitro assays, including a mix of W-Cu powders with standard grain size in the experiments to stablish comparisons. The effects of the direct exposure to powder suspensions and/or to their derived leachates were analysed in three model organisms representative of human and environmental exposures (the adenocarcinomic human alveolar basal epithelial cell line A549, the yeast *Saccharomyces cerevisiae* and the Gram negative bacterium *Vibrio fischeri*). Altogether, the results obtained provide new insights about the potential harmful effects of the selected nc alloys, showing that, from a toxicological perspective, nc TiAl is the safest candidate in the model organisms and conditions tested.

## Introduction

The concept of nanocrystalline (nc) materials, which was introduced for the first time by Gleiter^1^, in the specific case of alloys, is referred to a metal presenting a mean grain size in the nanometer scale, being this value below 100 nm. The extraordinary mechanical, chemical and physical properties that these materials present have attracted the attention of the scientific community and, therefore, nc metals have been the object of intense interdisciplinary research^2–6^. Among the outstanding characteristics that these materials possess, it can be highlighted their exceptional catalytic and thermal features, as well as their great strength, hardness and enhanced wear resistance^7^, all of them given by their specific structural properties. Thus, the use of nc alloys could represent a significant impact in those sectors where there is a need for the application of materials with these special features, such as the aerospace or aeronautical industries.

In the last years, and prompted by the strong competition that exists in the field, the development of new materials for aeronautic applications has been considered one of the main priorities for this sector^8^. Thus, extensive research has been conducted to address this issue, putting the efforts on the search of materials that allow to reduce costs, while providing an improvement in their behaviour when subjected to hard conditions (wear and corrosion resistance, damage tolerance…)^9–11^. Hence, elements selection to develop a new candidate alloy is a critical step that should be carefully considered. In this context, the mechanical alloying technique, which allows for the combination of elements that are difficult or impossible to melt by other conventional procedures^12^, outstands as a relevant methodology for the production of new alloys. Moreover, in the case of metallic nanomaterials, alloying has been proved to significantly enlarge the temperature range where coarsening is inhibited, which is one of the detrimental effects that these materials might present when they are constantly exposed to high temperatures^4,13,14^. In this regard, several nc metallic alloys have shown an improved behaviour at high temperatures as compared with their pure metal counterparts^15,16^.

In addition to exceptional mechanical and physical characteristics that newly developed alloys should have to allow their use in the areas of interest, their human and environmental safety should be considered. In fact, the toxicity of different relevant metal alloys widely employed in different fields such as biomedical or military (fabrication of medical devices, ammunition manufacturing…)^17,18^ has been already evaluated using both in vitro and in vivo methods^19–22^. However, with the rise of the additive manufacturing industry over the last years, the use of metal powders is currently much more widespread. Since metal powders can result more toxic than their bulk counterparts^23^, the provision of information about possible consequences associated to their handling and management must be considered of critical importance, considering as well potential leaching due to their deterioration and degradation.

In this study, three binary nanograin type metal alloys produced by mechanical alloying, with promising characteristics in terms of performance levels under extreme conditions and feasibility of fabrication, were evaluated from a toxicological perspective. The following binary alloys were selected: one lightweight heat-resistant high-strength metal alloy for aeronautical applications: TiAl; and two radiation-resistant metal alloys for radiation-shielding space applications: WAl and WCu. Hence, the biological impact of the alloy powders of nc WCu, WAl and TiAl was analysed performing different in vitro assays using two model organisms that were selected as representatives of human (A549 cell line) and environmental exposures (*Saccharomyces cerevisiae*). In addition, the potential toxicity of the alloy leachates produced by these materials was studied in the A549 cell line, as well as in the bioluminescent bacterium *Vibrio fischeri*. A standard grain size commercial grade W-Cu powder mix (25 wt% Cu) was also included in the experiments. Altogether, this work provides preliminary information about the safety of the different nc alloys, giving valuable data to help to determine the most appropriate candidate considering their potential hazardous properties.

## Results and discussion

### Characterization of the alloys powders and their associated leachates

Three binary nc alloys have been selected in the present study for their toxicological evaluation, due to their special features and potential application in the aeronautical industry. The properties of different nc WAl alloys, including a W_80_Al_20_ as the one studied in this work, have been described recently, showing that the addition of Al resulted in a significant improvement of coarsening resistance and sinterability with respect to pure commercial tungsten^24^. Regarding nc WCu, the characteristics of this alloy were also recently investigated^25^. W and Cu are immiscible metallic systems that are alloyable by the application of mechanical alloying, combining the excellent attributes of the W (high thermomechanical and radiation shielding features) with those of the Cu (high thermal and electric conductivity)^12,26^. Finally, TiAl is an intermetallic phase where Ti and Al are bonded by stoichiometric constraints. Ti alloys are widely applied for the fabrication of structural components for aircrafts, since they present exceptional strength as well as corrosion resistance^8^.

Composition, crystal structure, particle size and leachability are factors that can influence the toxicity of alloy powders, being SEM, XRD and ICP-MS very useful to evaluate these parameters. Therefore, the alloy powders studied in this work were characterized applying the mentioned methodologies. First, the average crystallite size of the three nc alloys (ncWCu, WAl and TiAl) was calculated using the Scherrer equation. This formula, which is effective for nano-sized crystallite, is based on the XRD patterns acquired on the mechanically alloyed powders (supplementary material, Figs. S1, S2, S3). The average grain size of each sample is represented in Table 1, which shows that the three studied nc alloys exhibited similar values.

**Table 1:** Average grain size in the nc alloys.

Sample	Average grain size (nm)
*ncWCu*	≈ 11
*WAl*	≈ 9
*TiAl*	≈ 10

**Figure 1:**
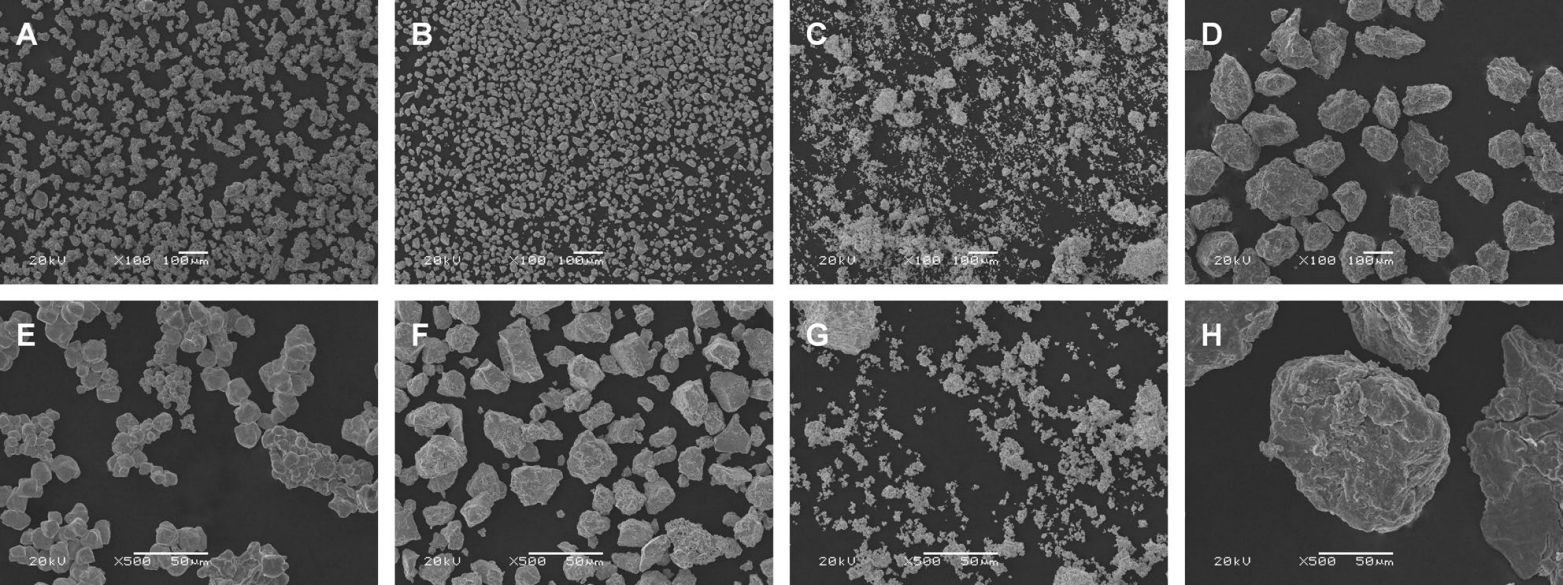
Morphology of the metal powders. sWCu (**A**), (**E**); ncWCu (**B**), (**F**); WAl (**C**), (**G**) and TiAl (**D**), (**H**). Images (**A**), (**B**), (**C**) and (**D**): Original magnification × 100 (Scale bar = 100 μm); Images (**E**), (**F**), (**G**) and (**H**): Original magnification × 500 (Scale bar = 50 μm).

SEM and SEM EDX were respectively used to visualize and analyse the elemental composition of all the alloy powders (wt%), including those from the standard grain size WCu (sWCu) sample. Since carbon adhesive tape was used in the preparation of the samples, low percentages of C were detected in all of them (≤ 8%), as well as low percentages of O, which are indicative of low degree of oxidation in the surface. In addition to these elements, low levels of others related with the materials of the milling means applied, such as Ni or Fe, were detected in the samples. Since this sample was produced by the mixture of the powders of the 2 elements, the sWCu, as expected, showed to be heterogeneous. Thus, different particles of W and Cu were detected, and also distinguished by scanning electron microscopy (Fig. 1). Regarding ncWCu, the percentages of W and Cu detected were 59.13% ± 1.99 and 26,37% ± 1.02 respectively. In case of WAl and TiAl samples, the percentages of the different elements detected were 89.18% ± 1.80 of W and 3.78% ± 0.43 of Al in the former, and 77.18% ± 3.24 of Ti and 13.95% ± 2.23 of Al in the later.

The morphology of the selected samples was analysed and visualized by SEM (Fig. 1). In all cases, a variety of particles, with sizes on the micrometer scale, mostly from a few to several dozens of micrometers, were observed. TiAl powders showed the biggest sized particles (Fig. 1D,H), while WAl particles showed the highest heterogeneity (Fig. 1C,G). Concerning their shape and morphology, polygonal and round particles were distinguished in all nc alloys. In case of sWCu, W particles showed to be rounded, forming structures that resemble aggregates, and smaller than Cu particles, which showed more angular shapes (Fig. 1A,E).

Furthermore, the presence of the elements composing the selected powders was evaluated in leachates obtained after incubating the materials in water, at a concentration of 10 g/L, for 3 months. W and Cu levels were analysed in the sWCu and the ncWCu samples. The nc alloy showed to release higher concentrations of both elements (W: 323,669.15 ppb; Cu: 45,110.10 ppb) in comparison with the standard grain size sample (W: 119,101.26 ppb; Cu: 4096.84 ppb). In the case of the nc WAl leachates, lower levels of W than those observed in both WCu samples were detected (24,829 ppb), while the detected Al concentration was low (136.31 ppb). Finally, the TiAl alloy showed to be less likely to leachate in water, since the Ti and Al levels detected were very low (Al: 11.27 ppb; Ti: 0.23 ppb) in comparison with those measured for their correspondent elements in the other alloys.

In summary, the physico-chemical characterization analyses revealed that the three studied nc alloys have similar grain size values, being all of them formed by particles in the micrometer scale, with variable leachability.

### Evaluation of metal powders and their leachates toxicity in the A549 human cell line

**Figure 2:**
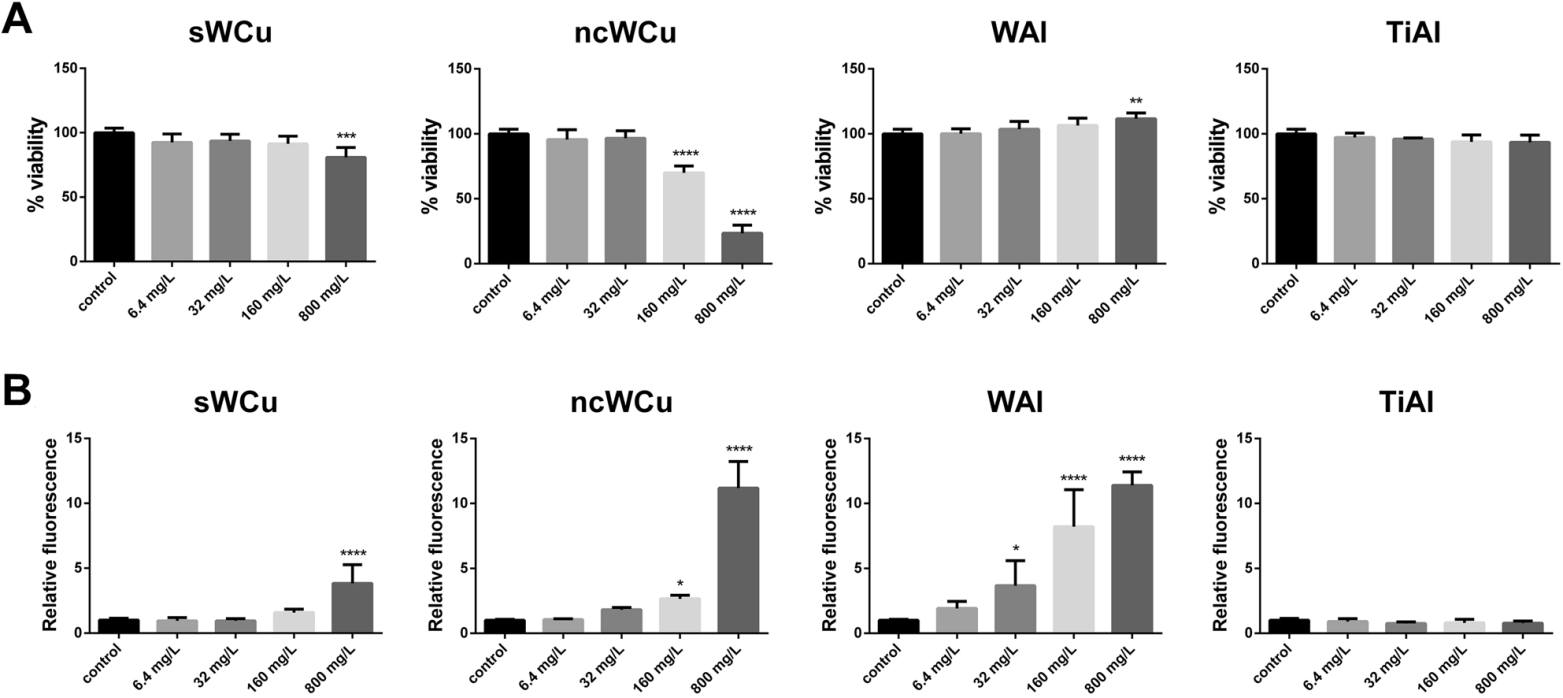
Impact of direct exposition of A549 cells to different concentrations of the metal powders. (**A**) Viability of A549 cells (Neutral Red assay). Results are expressed as % of control (untreated cells). (**B**) Oxidative stress (ROS levels) in A549 cells. Results are expressed as the relative fluorescence value to the control (untreated cells) which was assigned a value of 1. Data represent the mean ± standard deviation, SD. Differences were established using a One-way ANOVA followed by Dunnett post hoc test to compare every mean with the control, and considered significant at *P* ≤ 0.05. *P ≤ 0.05, **P ≤ 0.01, ***P ≤ 0.001, ****P ≤ 0.0001.

The A549 cell line was used as model of human exposure to study the possible hazardous effects of the nc alloys. Thus, the viability of this cell line after being exposed to different concentrations of the metal powders, as well as the ability of these materials to trigger oxidative stress, were evaluated. The results obtained in the viability test, which was analysed performing the neutral red uptake assay, are presented in Fig. 2A. A statistically significant decrease in the percentage of viable cells was observed after their exposure to 160 and 800 mg/L of ncWCu powders during 24 h, showing viability values of ≈ 70% and ≈ 20% respectively. In case of the standard grain size sample, only a significant decrease in this parameter was observed when cells were exposed to 800 mg/L (≈ 20%). Regarding the A549 cells exposed to WAl and TiAl nc alloy powders, no negative effect on their viability was observed, indicating that both materials are safe for this model organism in the conditions tested.

The ability of the selected materials to induce oxidative stress was also assessed in the A549 cell line using the DCFH-DA as indicator of ROS production (Fig. 2B). The obtained results show oxidative stress production in A549 cells after 1 h of exposure to ncWCu powders, being the observed ROS levels statistically significant at the two highest concentrations tested (160 and 800 mg/L). However, cells exposed to sWCu showed statistically significant levels of oxidative stress only when they were incubated in the presence of 800 mg/L, having around two times lower ROS levels than those induced by ncWCu. For their part, WAl powders also increased significantly A549 cells ROS levels, in a clear dose dependent manner, starting from 32 mg/L. In contrast, TiAl powders showed no ability to induce oxidative stress in A549 cells, in the conditions tested.

**Figure 3:**
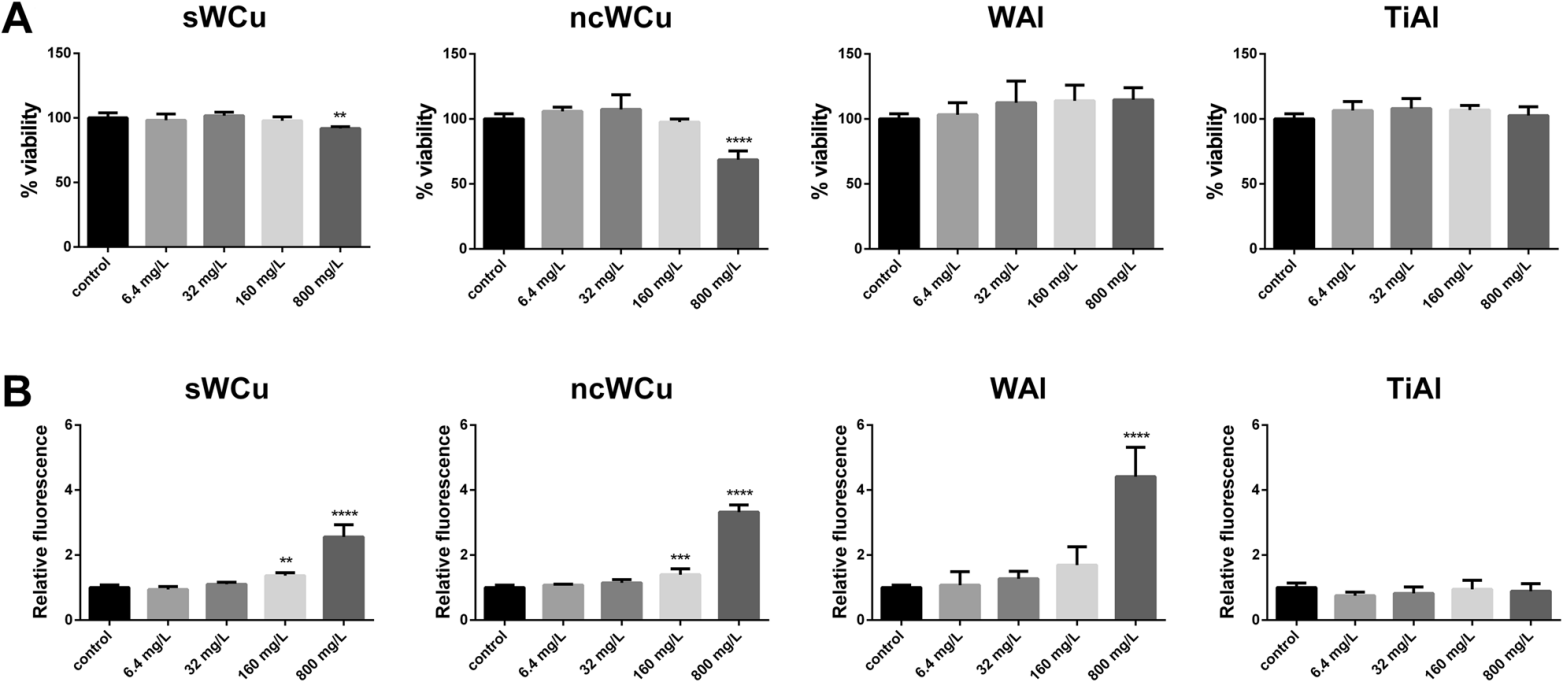
Effects in A549 cells after being exposed to different concentrations of the powder leachates. (**A**) Viability of A549 cells (Neutral Red assay). Results are expressed as % of control (untreated cells). (**B**) Oxidative stress (ROS levels) in A549 cells. Results are expressed as the relative fluorescence value to the control (untreated cells) which was assigned a value of 1. Data represent the mean ± standard deviation, SD. Differences were established using a One-way ANOVA followed by Dunnett post hoc test to compare every mean with the control, and considered significant at *P* ≤ 0.05. **P ≤ 0.01, ***P ≤ 0.001, ****P ≤ 0.0001.

The toxicological potential of the leached products obtained after the incubation of the metal powders during 3 months in water was also evaluated through the same assays employed to perform the direct contact tests. As displayed in Fig. 3A, only the leachates concentrations equivalent to 800 mg/L of both WCu samples decreased the viability of the A549 cells. This effect was higher in the case of the cells exposed ncWCu leachates, which showed a reduction of ≈ 30% in their viability, while sWCu leachates caused a decrease of $\approx$ 10%. WAl and TiAl alloy leachates showed to be safe in all the conditions tested. Regarding their ability to induce oxidative stress (Fig. 3B), both WCu leachates showed to induce statistically significant ROS levels at concentrations equivalent to 160 and 800 mg/L. In case of WAl leachates, significant oxidative stress levels were only observed at concentrations equivalent to 800 mg/L. Finally, as it was observed in the direct contact determinations for TiAl, leachates from this material did not induce oxidative stress at any of the concentrations tested.

The widespread use of metal alloys in a variety of fields has made necessary their toxicological evaluation, where different cell lines have been employed as model organism. For example, the biocompatibility of Ti alloys applied in the fabrication of medical devices, such as dental or orthopaedic implants, has been extensively studied using distinct human cell lines obtained from different tissues^27^. By the same token, the toxicity of W alloys, with an increased use in military applications substituting lead in ammunition fabrication, has been subject of study through the use of a variety of cell lines^28^. Besides the composition, size and form are important factors that critically affect the inherent toxicity of an alloy. For instance, in the case of Ti, experimental results showed that the elemental powder of this metal can be substantially cytotoxic, while bulk Ti is biocompatible^23^. The authors explained the observed differences arguing that the concentration of ions released into the media by bulk elemental metals is significantly lower than that released by powder, thus being less cytotoxic. In the present work, the selected alloys subjected to study were in powder form, presenting particles in the micrometre scale. Considering their ability to reduce cell viability and cause oxidative stress, the results showed that ncWCu is the most cytotoxic alloy, while TiAl is the safest one in the studied conditions. The obtained results also indicated a correspondence between the susceptibility of the materials to leach and their associated toxicity: ncWCu was the alloy releasing the highest levels of its components in the leachates, while TiAl leached very low levels of Ti and Al. In addition, the lower levels of Cu and W detected in the sWCu leachate, compared with those detected in its alloyed counterpart (W and Cu concentration was ≈ 3 and ≈ 11 times higher in ncWCu leachates than in sWCu leachates, respectively), could also explain their differences in toxicity over A549 cells. In this regard, Palombella et al. analysed the cytotoxic potential of micro and nanoparticles of zero-valent iron, cobalt, and nickel in human adipose stem cells, suggesting that the pernicious effects that the microparticles produced could be caused by the release of ions in the surrounding medium, or by the presence of these particles around the cells, which would led to a reduction in the oxygen and nutrient exchange efficiency^29^.

Considering that all the leachates analysed in this work, except those from TiAl (having very low Ti and Al concentrations), can cause oxidative stress and even cell death (in case of both WCu samples), it can be suggested that the released ions play an important role in the toxicity of the studied materials. Moreover, since the intensity of the cytotoxic response was higher in the direct contact experiments, it can be assumed that the observed effects are produced by a combination of the released ions together with the direct interaction of the cells with the metal particles. In this regard, it can be highlighted that some metal alloy particles have been described to cause oxidative stress without the need of releasing hazardous levels of ions to the medium^30,31^.

### Evaluation of metal powders toxicity in the yeast *Saccharomyces cerevisiae*

**Figure 4:**
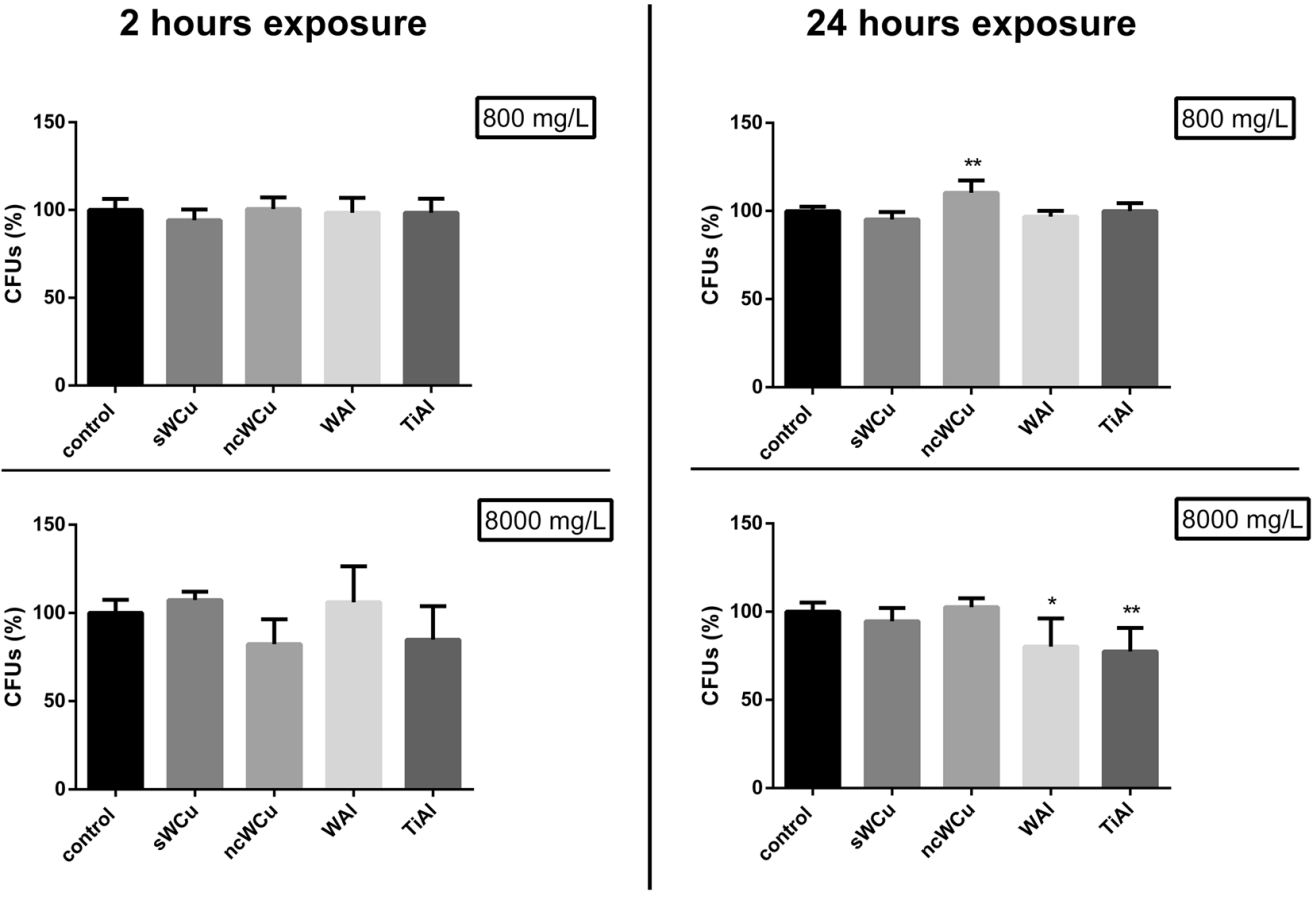
Colony forming units (CFUs) of *S. cerevisiae* cells exposed to different metal powders at two exposure times (2 and 24 h) and two concentrations (800 and 8000 mg/L). Results are expressed as the percentage (%) of CFUs determined for each exposure condition using as reference value the non-exposed cells condition, which was assigned a value of 100%. Data represent the mean ± standard deviation, SD. Differences were established using a One-way ANOVA followed by Dunnett post hoc test to compare every mean with the control, and considered significant at *P* ≤ 0.05. *P ≤ 0.05, **P ≤ 0.01.

The toxicological potential of the nc alloys with different composition was also determined for the yeast *Saccharomyces cerevisiae*, an amenable unicellular fungus widely used as model to understand molecular mechanisms in eukaryotic cells^32^, and in ecotoxicology studies too^33^. The viability of yeast cells after exposure to two different materials concentrations (800 and 8000 mg/L) and times (2 and 24 h) was analysed through colony forming units (CFUs) determination (Fig. 4).

At short exposure time (2 h), no significant differences in *S. cerevisiae* viability could be observed between the control condition (non-exposed cells) and any of the 4 samples tested at both concentrations. However, when the exposure time was increased (24 h), some viability differences could be observed amongst the studied conditions. In particular, a significant decrease in the viability of *S. cerevisiae* cells was observed in the presence of the higher concentration of WAl and TiAl alloys (*P* ≤ 0.01 and *P* ≤ 0.05 respectively) for 24 h.

The toxicological potential of the alloys present in liquid suspensions was determined as well investigating their ability to induce the formation of ROS. Again, 800 and 8000 mg/L of the different metal powders were used to expose *S. cerevisiae* cells, for 2 h.

**Figure 5:**
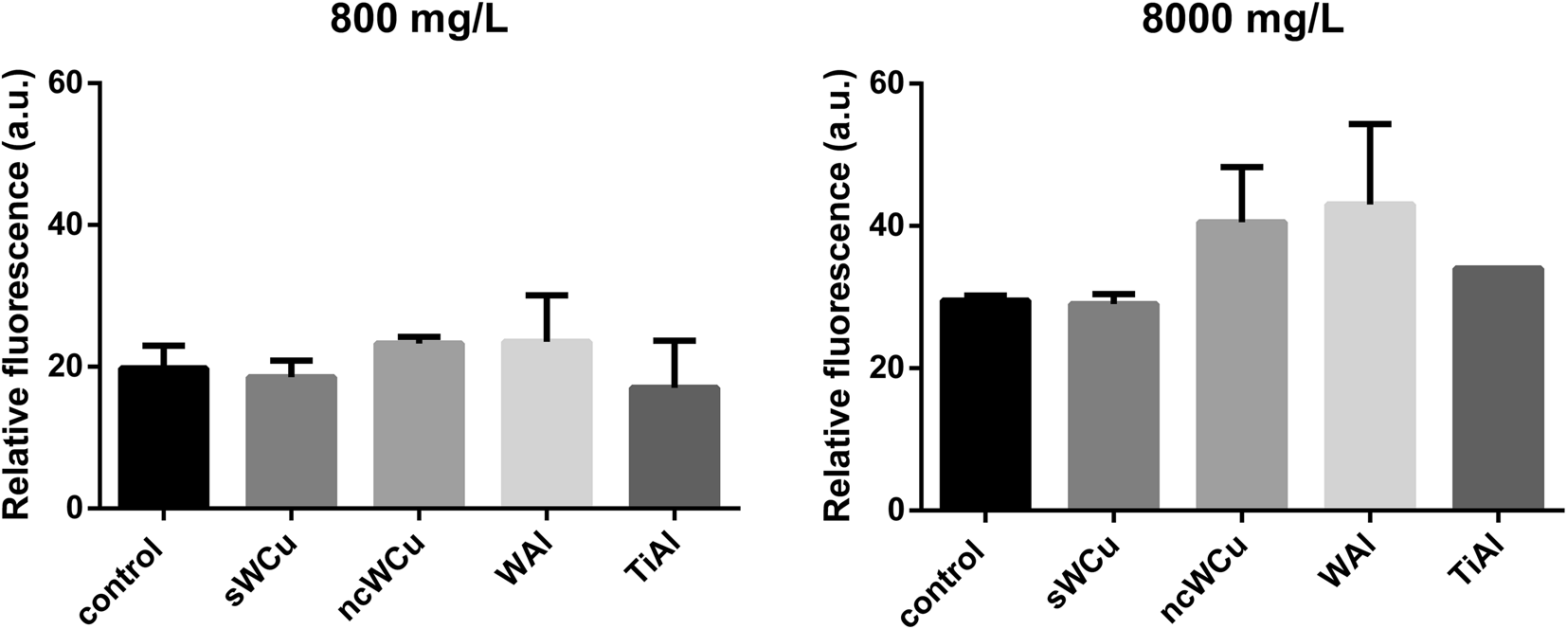
ROS induction analysis of *S. cerevisiae* cells exposed to different metal powders during 2 h at two different concentrations (800 and 8000 mg/L). Results are expressed as arbitrary fluorescence values. Data represent the mean ± standard deviation, SD. Differences were established using a One-way ANOVA followed by Dunnett post hoc test to compare every mean with the control, and considered significant at *P* ≤ 0.05.

As shown in Fig. 5, differences in relative fluorescence levels observed between the *S. cerevisiae* cells exposed to the distinct alloys were minor. At the highest concentration, the highest average ROS levels were observed for WCu and TiAl alloys, but they were not significantly different than those observed for the rest of the conditions tested, including the control condition.

While the effect of metals and metalloids on *S. cerevisiae* has been a topic of study for many researchers, generating knowledge in different aspects of metal biology^34^, little is known about the effect on fungi and other microorganisms of specific metal combinations. The obtained results indicate that the ability to induce cellular damage by the different alloys in *S. cerevisiae* cells, even in the presence of very high concentrations, is very low. This indicates that factors governing metals toxicity in *S. cerevisiae*, such as the concentration, speciation and oxidation state are of minor concern in case of the alloys under analysis. Therefore, due to the low observed toxicity, we decided not to perform an additional analyses using alloys leachate solutions.

### Evaluation of metal powders leachates toxicity in *V. fischeri*

**Figure 6:**
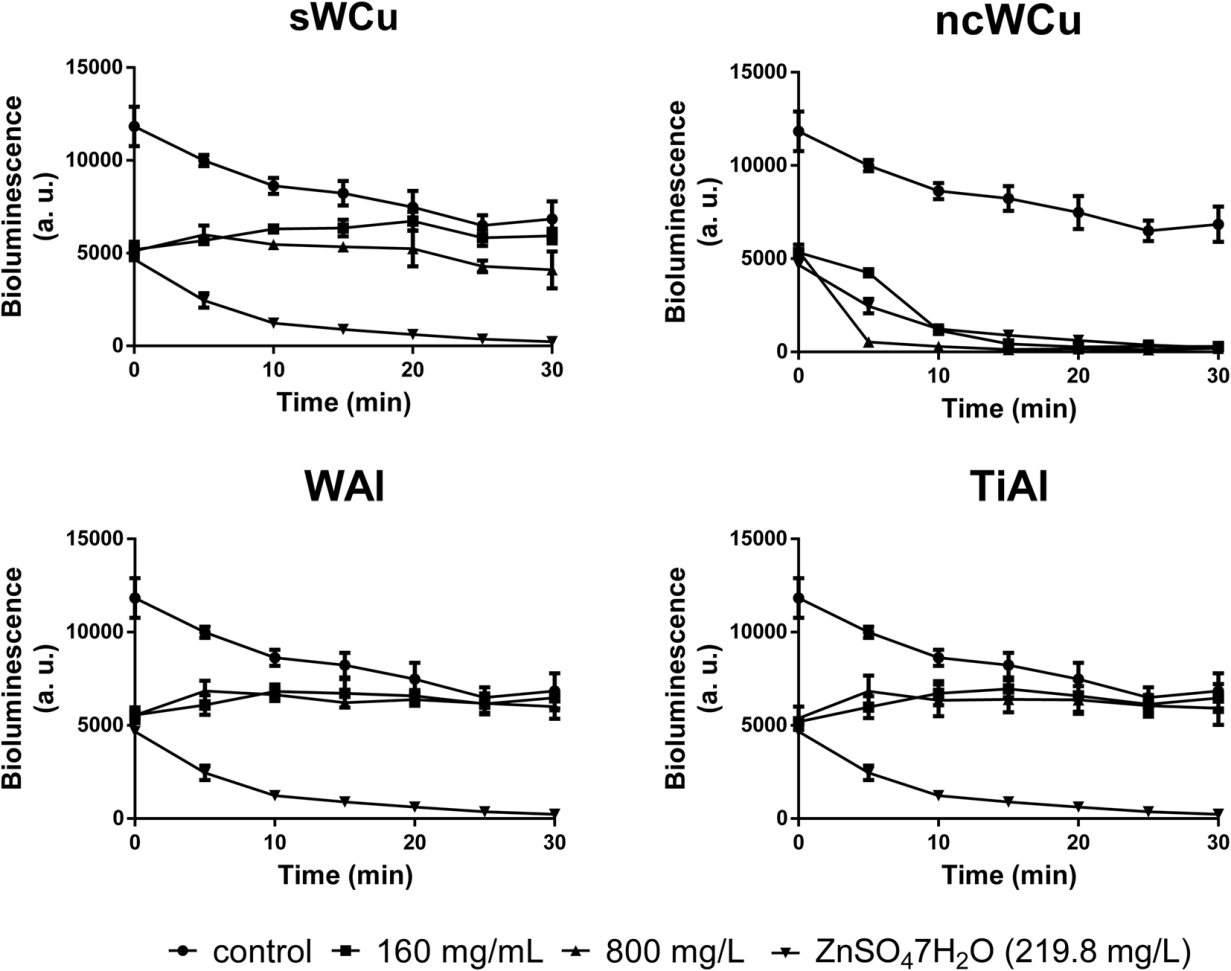
Impact of the different metal powder leachates in the bioluminescence of *V. fischeri* during a 30 min-exposure.

The impact of the nc alloys in the marine bacterium *V. fischeri* was evaluated monitoring the bioluminescence produced by this microorganism in presence of leachates equivalent to 160 and 800 mg/L of the different materials. Figure 6 represents the evolution of the light intensity produced by the bacteria monitored over a 30-min period with intervals of 5 min. Curves showed that all the leachates caused a drop of ≈ 50% in the initial bioluminescence peak, similar to the drop observed in the inhibition control (ZnSO_4_·7H_2_O). From this point on, the bioluminescence remained constant in the bacterial suspensions exposed to WAl and TiAl leachates, being the light intensity comparable to that shown by the control (non-exposed cells) for both concentrations tested after 20 min of exposure. In the case of the bacteria incubated with leachates equivalent to 160 mg/L of sWCu, the evolution of the light intensity was similar to that described for TiAl and WAl leachates, while a decrease in the bioluminescence was observed at concentration equivalent to 800 mg/L after 30 min of incubation (≈ 40% less luminescence than in the control). Leachates from ncWCu showed to have a critical impact in *V. fischeri*, as the two concentrations used to perform the experiment provoked a drop in the light intensity similar to that observed in the inhibition control (ZnSO_4_·7H_2_O), leading to a complete loss of bioluminescence.

Due to its simplicity and rapid response, together with the reliability and sensitivity of the tests where it is applied, the marine bacterium *V. fischeri* is a useful organism to study the toxicity of different compounds^35^. For instance, this bioluminescent organism has been applied to evaluate the potential hazard of different heavy metals and mixtures of them^36^, as well as in combination with other substances, such as polycyclic aromatic hydrocarbons or humic acids^37,38^. In relation to the marine bacterium exposure to different metallic leachates, our results have shown that those derived from ncWCu, containing very high concentrations of Cu and W, reduced dramatically its light intensity at both concentrations tested. Previously, it was reported that 0.8 mg/L of Cu was able to induce an almost full inhibition of the light emitted by *V. fischeri* after 2 h of exposure^37^. Therefore, the very high concentration of this element detected in the ncWCu leachate was probably the main cause of the observed effects. While, Cu and W were also found in the leachates obtained from sWCu powders, the concentrations of both elements were much lower, so the decrease in *V. fischeri* luminescence was only observed in the exposure to the highest concentration tested.

## Conclusions

The potential impact that a newly developed material may represent for the human health and the environment is an issue that should be properly considered during its developmental stage. In the present study, three nanocrystalline binary alloys considered as promising candidates for their use in the aerospace industry, due to their challenging properties under extreme conditions and feasibility of fabrication, were evaluated from a toxicological perspective. Thus, metal powders of WCu, WAl and TiAl nc alloys and their associated leachates were analysed in different model organisms. In addition, a powder sample containing a standard grain size W-Cu mixture was included in the assays. In general terms, TiAl stood out as the safest alloy, since only huge concentrations of the powders resulted in negative effects for yeast cells, while neither the leachates, nor the powders, produced harmful effects in the rest of the organisms tested, at none of the concentrations evaluated.

A relationship seems to exist between the toxicity of the alloys and their susceptibility to leach elements to the aqueous medium. While very low levels of Ti and Al were leached from the TiAl nc alloy, which resulted to be the safest material of those tested in the present study, ncWCu showed a great susceptibility to leach, being as well the most toxic one for A549 cells and *V. fischeri* in the conditions tested. Surprisingly, this alloy showed to be harmless for yeast, where even at huge concentrations did not cause neither a decrease in its viability, nor oxidative stress. Altogether, the presented results provide a preliminary toxicological assessment of different nc alloys, indicating that, from a safety point of view, nc TiAl could be a good candidate alloy for its development and introduction in the industry.

## Materials and methods

### Synthesis of the alloys

The synthesis of nanocrystalline alloy powders used in this work to carry out toxicity assays was performed in the industrial plants of MBN nanomaterialia by High Energy Ball Milling, applying a proprietary mechano-chemical synthesis process technology (Mechanomade®). Industrial grade metal powder of W, Al, Cu and Ti were used in the right proportion to obtain the targeted composition. The systems W_50_Cu_50_, Ti_75_Al_25_ and W_80_Al_20_ were produced and named in this work as ncWCu, TiAl and WAl respectively. In order to avoid as much as possible the oxidation of the powder, the mechanical alloying process was performed in inert Ar atmosphere, which was also kept during the sieving performed to remove bigger particles, and during packaging. To diminish the contamination, the materials of the milling chamber and milling balls have been different for each binary alloy and for each milling equipment. In particular, the final materials used were the following:

W-Cu system: Bronze balls and hard steel for the milling chamber.

W-Al system: Hard steel for the milling chamber and the ball.

Ti–Al system: Titanium Grade 5 for the balls and hard steel for the milling chamber.

A mix of commercial grade Tungsten and Copper powders (25 wt% Cu) with standard grain size were also used in the experiments. This sample was named in this work as sWCu.

### XRD analysis of the powder samples

The microstructure of the mechanically alloyed powder samples was characterized by means of a SIEMENS 5005 XRD diffractometer, using Cobalt target, in the MBN nanomaterialia facilities.

### SEM–EDX analysis of the metal powders

The surface composition of the metal powders used in the toxicity assays were characterized semi-quantitatively applying SEM–EDX. A JEOL JSM-6460LV microscope equipped with a X-Max^N^ energy dispersive detector was used in the University of Burgos facilities to carry out the element identification and quantification of the metal powders. At least 3 different areas were randomly selected to perform the analysis of each material.

### Morphology analysis of the metal powders by SEM

The morphology and the size of the particles were visualized and analysed by Scanning Electron Microscopy. For this purpose, a small quantity of each sample was directly examined using JEOL JSM-6460LV at the University of Burgos.

### Preparation of the samples

Water suspensions of milled powders (10 g/L) were prepared, to be used in the direct contact experiments at different concentrations. Before each experiment, the metal powders stocks were homogenized vortexing the samples at full speed for 1 min, subjecting them to ultrasonication at low power intensity, and finally applying an additional vortex step.

The leachates were obtained by storing metal powders suspensions (10 g/L) at 4 °C during 3 months. Afterwards, the samples were centrifuged, and the supernatants were recovered and filtered through 0.22 polyethersulfone membranes.

### Leachates characterization by ICP-MS

The elements present in the sample leachates, obtained after the filtration with 0.2 µm polyethersulfone membrane filters of the aqueous fraction recovered from metal powders suspensions (10 g/L) incubated during 3 months, were studied by inductively coupled plasma mass spectrometry (ICP-MS) using an Agilent 8900 ICP-QQQ instrument in the University of Burgos.

### Model organisms used in the toxicological studies and culture conditions

A549 lung cancer cell line was cultured in commercial Dulbecco’s Modified Eagle’s Medium (DMEM) supplemented with 10% (v/v) fetal bovine serum (FBS) and 100 U/mL penicillin and 100 mg/L streptomycin. Cells were incubated at 37 °C in a humidified 5% CO_2_ atmosphere.

The BY4741 strain of *S. cerevisiae* was grown and maintained in standard YPD medium (1% yeast extract, 1% yeast bacto-peptone, 2% glucose). Cell cultures in liquid media were incubated on a rotary shaker at 185 rpm at 30 °C.

The bioluminescent Gram negative bacterium *V. fischeri* NRRL B-11177 was maintained at room temperature in Marine Broth or Agar 2216.

### Viability assay in A549 cell line

The viability of A549 cells directly exposed to nc alloy powders was evaluated applying the Neutral Red assay. Cells were seeded in 96 well plates in a density of 3 × 10^4^ cells per well and incubated 24 h after which cells were exposed to different concentrations of the materials (6.4, 32, 160 and 800 mg/L) resuspended in fresh treatment medium (DMEM with 1% FBS and without antibiotic). Cells incubated with treatment medium alone were included in the experiments as living cells control. After the exposure, wells were washed with DPBS, and 100 µL of a Neutral Red solution (40 µg/mL) were added to each well for 2.5 h. This solution was subsequently discarded, cells were washed once with DPBS and fixed for 2 min with formaldehyde 4%. Cells were washed again with DPBS, and 150 µL of a dye release solution containing 50% ethanol 96°, 49% distilled H_2_O, and 1% acetic acid were added to each well. Finally, plates were shaken for 10 min, and 100 μl of each well were transferred to an opaque 96-well plate to measure the fluorescence using a microplate reader (BioTek Synergy HT, excitation wavelength, 530/25; emission wavelength 645/40). Results were expressed as percentage of control (fluorescence of cells in absence of nc alloys). Two independent experiments were carried out, with 3 biological replicates per exposure condition in each case. To test the toxicity of the leachates, cells were exposed to different concentrations (leachates equivalent to 6.4, 32, 160 and 800 mg/L of the alloys) during 24 h, and cell viability was studied applying the above-explained protocol.

### Oxidative stress assay in A549 cells

A549 cells seeded in 96 well plates at 3 × 10^4^ cells per well were washed with Hank’s Balanced Salt Solution (HBSS) without phenol red, and subsequently incubated with a solution of DCFH-DA (2ʹ,7ʹ-Dichlorofluorescin Diacetate) in HBSS (50 µM) for 30 min at 37 °C in the dark. After this time, cells were washed again with HBSS, and then exposed to different concentrations of nc alloy powders (6.4, 32, 160 and 800 mg/L) resuspended in HBSS, using as control cells incubated with HBSS alone. Finally, fluorescence was measured after 60 min of incubation using a microplate reader (BioTek Synergy HT, excitation wavelength, 485/20; emission wavelength 528/20). Two independent experiments were carried out, with 3 biological replicates per exposure condition in each case. The oxidative stress induced by leachates equivalent to the same concentrations of the nc alloys used in the direct contact experiments was analysed applying the same protocol.

### Colony forming units (CFUs) determination applying *S. cerevisiae*

*S. cerevisiae* cells were pre-grown on YPD medium in an orbital shaker (30 ºC and 185 rpm) until an O.D.600 nm = 1 was reached, and then they were exposed to 800 or 8000 mg/L of the different metallic powders in the same medium culture, or cultured non exposed (negative control), in 24-well plates (final volume of 1 mL). Subsequently, culture samples were obtained after 2 and 24 h of exposure. To determine CFUs at both sampling times, 100 µL of cells were diluted 10^4^ times, in case of 2 h exposure, and 10^5^ times, in case of 24 h exposure, inoculated on solid YPD medium (6% agar) plates, and incubated at 30 °C for 48 h.

### Oxidative stress assay in *S. cerevisiae*

The CM-H2DCFDA dye was used to quantify intracellular ROS levels in yeast, employing a protocol similar to that previously described by James et al.^39^, recently adapted by our research group^40^. Briefly, *S. cerevisiae* cells growing in exponential phase were pelleted, washed and incubated with CM-H2DCFDA (7 µM) in DPBS for 60 min at 30 °C and 185 rpm. Subsequently, cells were washed again, resuspended in YPD and exposed to the different samples (800 and 8000 mg/L) for 2 h. Then, yeast cells were washed two times with DPBS, incubated 2 min in a solution containing Lithium Acetate 2 M, washed and incubated again for 2 min in a solution containing SDS (0.01%) and chloroform (0.4%). Cells were finally pelleted and the supernatant transferred to a black opaque 96 micro-well plate, where the fluorescence was measured (excitation wavelength, 485/20; emission wavelength 528/20) using a microplate reader (Synergy-HT, BioTek).

### Toxicological assay applying *V. fischeri*

The impact of the powder leachates, equivalent to 160 and 800 mg/L, on the luminescence produced by *V. fischeri* was studied applying the bioluminescence inhibition assay. Briefly, a 5 mL culture of Marine Broth 2216 inoculated with one luminescent colony was incubated for 48 h at 15 °C. The bacterial suspension was then centrifuged, and the pellet resuspended in 5 mL of NaCl 2% (w/v), and pre-incubated at 10 °C for 30 min before the start of the experiment. 90 µL of leachates resuspended in NaCl 2% solution at the concentrations specified above were added to each well in a 96 well opaque microplates. Non-exposed bacteria, resuspended in 2% NaCl, were included in the assay as control, to monitor the natural light attenuation of this microorganism, while bacterial cells incubated with ZnSO_4_·7H_2_O at 219.8 mg/L were included as light inhibition control. To carry out the experiment, 10 µL of the bacterial suspension were added into each well, and the plate was incubated in a Thermomixer (800 rpm, 15 °C). The luminescence of each sample was recorded in 5 min intervals throughout 30 min in a microplate reader (Synergy-HT, BioTek), being the starting point the value measured immediately after the addition of the bacteria.

### Statistical analysis

Data are presented as means ± SD. The one-way analysis of variance (ANOVA) followed by Dunnett post hoc test to stablish comparisons between every mean and the control was used. Statistical tests were carried out using Prism 6.0 (GraphPad Prism, GraphPad Software, Inc.), considering the differences significant at *P* ≤ 0.05.

## Supplementary Information

Supplementary Information.
