# Supplementary material for: Toxicological assessment of nanocrystalline metal alloys with potential applications in the aeronautical field
Source: Sci Rep. 2022 Jan 27;12:1523. doi: 10.1038/s41598-022-05406-5 (PMC8795356; doi:10.1038/s41598-022-05406-5)
Supplement: Supplementary file 1 — Supplementary Information. [file 41598_2022_5406_MOESM1_ESM.docx]

**

**

**Figure S1.** XRD pattern evolution of the ncWCu sample with increasing milling time.





**Figure S2:** XRD pattern of the WAl sample with long milling time.





**Figure S3:** XRD pattern evolution of the TiAl sample with increasing milling time.
